# 

**Published:** 1915-07-17

**Authors:** 


### CONTENTS.

| | PAGE |
|---|---|
| **War Service at Home** ... ... ... ... | 321 |
| **Hospitals and the Spirit Tax** ... ... ... | 322 |
| **Hospital and Institutional News** ... ... ... | 323 |
| The Control of Nursing Homes—St. Bartholomew's Hospital Staff and War Service—War Loan Gratuities—"Latest Installations in Hospital Equipment"—The Salaries of Health Visitors—Medical Attendance for the Soldier on Furlough—Margarine as an Economy—The Closing of Poor-Law Institutions—The Punishment of Refractory Insured Patients—The Poole Guardians and their Medical Officer—The Hospital Saturday Fund and Alien Instrument-Makers—A Reduction in a Drug Account—A Conference of Hospital Dispensers?—A New Delicacy in Sick-Room Cookery—A Farthing Fund—Alexandra Day at Cardiff—Not Wanted by the War Office—The Bacillus in the Book—A Case of Anthrax and a Shaving Brush—St. Andrew's Ambulance Corps—Children's Hospitals and the War—A Bad Night—This Week's Drug Market. | |
| **The Responsibility for the Medical Care of Hospital Patients** ... ... ... ... | 329 |
| **National Insurance Act Developments** ... ... | 330 |
| Disablement Pensions and Reduced Sickness Benefit. | |
| **Open-Air Wards in Peace and War** ... ... | 331 |
| The Lessons of the Cambridge Experiment. | |
| **Two Curious Bullet Wounds of the Neck** ... | 333 |
| By a Medical Officer at a Base in France. | |
| **Research and Remedies** ... ... ... ... ... | 332 |
| The Manner of Infection in Polio-Myelitis—Traumatic Aneurisms—An Obstetric Accident. | |
| **The Educative Work of the School Doctor** ... | 334 |
| Nasal Troubles in School Children. | |
| **Legal Notes for Institutional Workers** ... | 335 |
| A Panel Doctors' Fees: Judgment on Appeal—Accident to a Hospital Patient—A Hospital Employee's Negligence. | |
| **Round the World** ... ... ... ... ... | 336 |
| Fellow-Workers and the Roll of Honour. | |
| **The Laundry Expenses of Hospitals** ... ... | 337 |
| King Edward's Hospital Fund Statistics. | |
| **Oldham's Work for the Wounded** ... ... | 339 |
| **The Editor's Letter-Box** ... ... ... ... | 340 |
| How THE HOSPITAL is Helping in the War—A Reduction in a Drug Account—The Hospital Egg: An Egg-Farmer's View. | |
| **Institutional Needs** ... ... ... ... ... ... | iv |
| **Personalia** ... ... ... ... ... ... ... | iv |
| **Buildings Contemplated and in Progress** ... | iv |
| **Latest Installations in Hospital Equipment** ... | iv |
| **The Institutional Worker** ... ... (Suppl.) | 1 |
| The Admission of In-Patients: Answer to June Question. | |
| Question for July. | |
| Questions Invited. | |

